# Stochastic Identification of Stability of Competitive Interactions in Ecosystems

**DOI:** 10.1371/journal.pone.0155023

**Published:** 2016-05-12

**Authors:** Marek Vach, Pavla Vachová

**Affiliations:** Faculty of Environmental Sciences, Czech University of Life Sciences Prague, Kamýcká 129, Praha 6 Suchdol, 165 21, Czech Republic; University of Waterloo, CANADA

## Abstract

The problem of finding an optimum within a set of possibilities that represent the varying successfulness of numerous subjects competing with one another is highly relevant in the field of ecosystem interactions. We propose a method for solving this problem by the application of the Nash equilibrium concept, which is frequently used in ecology. The proposed model is based on the transformation of the initial payoff vectors of subjects that interact in different situations into a statistical set of symmetrical game matrices that consist of permutations of payoff values. The equilibrium solution is expressed as values of the probability of Nash equilibrium occurrence with uniform distribution over all possible permutations based on uncertainty of positions of payoff values in the matrix. We assume that this equilibrium solution provides information on the distribution of the degree of stability among individual situations and interacting subjects. In this paper, we validate this assumption and demonstrate its application to a dataset that represents interspecies interactions in plant ecology. We propose that the use of the Nash equilibrium in the analysis of datasets formalized according to the Pareto optimality scheme is applicable in numerous other contexts.

## Introduction

The problem of finding an optimum within a set of possibilities that represent the varying successfulness of numerous subjects competing with one another is not only a highly relevant basic topic in the field of economics but also in other disciplines. The basic approach for solving this problem in economics is to search for the Pareto optimality (PO) that corresponds to the state in which no subject can achieve a better result without worsening the result of another subject. The PO problem is often encountered in economics research. Of recently published research, the paper of Luc [1] is notable. The paper provides a detailed, exact analysis of a PO problem. The PO concept is practical, and it is frequently used in many contexts including environmental sciences [2–5]. Nevertheless, an evaluation of the equilibrium optimum within the set of possibilities that represent various successful competitive environments, e.g., the species represented in an ecosystem, may be realized using other approaches. In the first place, the application of the Nash equilibrium (NE), which is a fundamental principle of game theory [6], is offered as an option. Additionally, both principles—PO and NE—may be compared within the framework of the matrix game scheme. PO can be found in states that are not necessarily identical with NE [7].

In this paper, we propose a method for solving the problem of the equilibrium optimum in a set of situations with various payoffs for interacting subjects by applying the NE concept, which is frequently used in ecology. Most cases that involve the application of advanced game theory are referred to as evolutionary game theory (EGT). In the theory of evolutionary processes, game models are viewed as the basic unit of frequency-dependent interspecies interactions. Basic interaction types (e.g., plant–plant competition, plant–herbivore interactions, and plant–mutualism interaction [8]) are modeled based on defined strategies that species acquire during evolutionary development.

When simulating adaptive dynamics in phenotype space, a game model of interactions is applied in the replicator equation, the solution to which provides a picture of short- and long-term evolution [9]. The simulated population’s per-capita growth rate can be determined using the G-function [10]. This function, which includes a specific game model, is dependent on the sizes and strategies of the interacting populations as well as the particular strategy, i.e., a trait of the species whose evolutionary development is being simulated [11, 12].

In EGT, the attainment of an evolutionarily stable strategy (ESS) is viewed as the optimal solution [13]. Every ESS is a strategy in a Nash equilibrium in the stage of linear game, although not all NE are made up of ESS. Matrix games as models of evolutionary interaction have been applied in many studies [14–20]. EGT has been applied to a number of specific cases, such as adaptations by organisms to recent climate changes [21], competitive interactions between root systems of plants [22], and many others. An example of an EGT-based study is the application of advanced game theory to the coordination of ecosystem management strategies [23]. In this example, the replicator equation follows from a bioeconomic game model, and the stable state is a NE.

In sum, the practical application of game theory in environmental science results in interactive models in the form of game types of various degrees of complexity. However, in contrast to the game concept, the problem analyzed in this research, i.e., the search for the equilibrium optimum within a set of variant situations that represent various payoffs for interacting subjects, does not include a deterministic selection of strategies. The problem that we solve may be regarded as a purely stochastic one. We propose a model for the evaluation of the equilibrium optimum in a set of observed data. In principle, this evaluation may be considered analogous to statistical data processing.

The proposed approach is based on transformation of the initial payoff vectors of subjects who interact in different situations in a statistical set of symmetrical game matrices that consist of permutations of payoff values. Additionally, the concept of variable payoff vectors in game models is introduced within a framework of the inclusion of more types of possible behavior of individual players. However, this proposed approach does not exclusively concern a stochastic problem. The result of the game is determined by the selection of strategies even though these strategies are linked with a specific type of player behavior defined by a probability value. Thus, extended game models were established by Harsanyi [24, 25] and have been developed particularly in the field of cooperative game theory [26, 27] and related research.

The solution proposed in this paper also transcends the category of stochastic games formed by a sequence of random states. The actual payoff depends on previous states of the game. Stochastic games were introduced by Shapley [28].

Our proposal for solution of the equilibrium optimum in a set of various situations is based on permutation of *N*-plets of payoff values of *N* interacting subjects in a symmetrical game matrix of a corresponding dimension. The individual variant configurations of this matrix are fully independent. That is, they do not exhibit a relative structural connection. Each incurred matrix represents a linear game in normal form with at least one NE. Therefore, the substance of the proposed model consists of the equilibrium solution of a symmetrical game matrix derived from payoff values of subjects that interact in a set of various situations. This equilibrium solution is expressed as values of the probability of NE occurrence with uniform distribution over all possible permutations given by the uncertainty of the positions of *N*-plets of payoff values in the matrix.

We assume that this equilibrium solution provides information on the distribution of the degree of stability among the individual situations and the interacting subjects. The problem of ecosystem stability is the topic of many works, where the relationship between stability and diversity is often evaluated [29–34]. We propose a stochastic model for calculation of the degree of stability that can be used to evaluate e.g. the selected data sets of ecosystem descriptors. Our approach can be applied in the simplest case to the numerical example of two species observed at four sampling sites mentioned in Chapter 4 of Legendre and Legendre Numerical Ecology [35]. A similar scheme is of course addressed in many research works, not only in ecology.

## The model

Let a set of *K* alternative situations be defined by different external conditions. In each *K* situation, *N* subjects interact with one another. The results of this interaction are expressed by payoff values for each subject in all *K* situations. That is, the individual situations are advantageous to different degrees for the interacting subjects. The aim is to find the continuous distribution of individual situations along an axis that represents the equilibrium determined by a total degree of advantageousness for all interacting subjects. The maximum degree of equilibrium will be displayed by the situation that provides the best possible compromise of payoff values for all subjects. However, the evaluation of equilibrium is based on the NE concept, which cannot be found in a set of individual vectors of payoff values. Therefore, for the proposed method of evaluation, it is necessary to transform the initial payoff vectors into a normal form game matrix of dimension *N*. However, no information is available on the possible strategies of the interacting subjects that could determine the particular configuration of the game matrix. Nevertheless, it is possible to define two basic, apparent conditions for this transformation of payoff vectors into the game matrix. The first starting principle is that the derived game matrix should be symmetrical with respect to the number of interacting possibilities—therefore, formal strategies of interacting subjects in the sense of defining the game in the basic form. The second condition is connected with the stochastic substance of the proposed approach, which can be expressed as follows. Any information that substantiates a particular form of game matrix for NE evaluation is unavailable. Then, the only correct possibility is to include a complete set of all possible forms of game matrices to be derived, which can differ depending on the NE position within the limits of the elements of the game matrices. Of course, the elements in the derived matrices correspond to payoff values of *N* subjects in *K* individual situations $\pi_{1}^{k},\;...\pi_{N}^{k}$. Permutations that result in the formation of individual game matrices are identical in relation to interacting subjects (i.e., players). The individual game matrices concern permutations of *K* situations with fixed payoff values for players $\pi_{1}^{k},\;...\pi_{N}^{k}$, which are invariable with respect to changes in position in the matrix. The maximum, which is represented by a complete set of all possible permutations that determine the occurrence of NE in derived games, is achieved in the symmetrical payoff matrix of dimension *N*. If strategies are not made concrete, i.e., if (considering the evaluated NE position of the elements in the matrix) the sequence of columns or rows of the matrices to be derived does not matter because the total number of available games *V* with the possibility of a different NE result for *N* subjects (players) is given as follows:

$$\begin{array}{c} V=\frac{(\prod_{n}^{N}A_{n})!}{\prod_{n}^{N}A_{n}!} \end{array}$$ (1)

An is the number of formal strategies of player *n*. The number of possible permutations *V* of the payoff matrix is the maximum for *A*_1_ = *A*_2_ = … = *A*_*N*_; therefore, for a symmetrical game matrix of dimension *N*. Because $(\prod_{n}^{N}A_{n})!=K!$ is a constant and for the denominator is: $(A+\tau )!\cdot (K/(A+\tau ))!>A{!}^{2},A=\sqrt{K},\tau \neq 0$ for a pair of interacting entities.

The evaluation of equilibrium for payoff vectors $\pi_{n}^{k}$ primarily requires their linear mapping *φ* : *X*_*K*_ → *Y*_*K*′_ from space of variant situations *X*_*K*_ into space *Y*_*K*′_, dimension *K*′, which will enable the formation of symmetrical matrices, i.e., *K*′ *ϵ* *A*^*N*^, where *A* > 1 is the selected number of columns and rows of the game payoff matrix. For example, the outcome for two interacting subjects may be *K*′ = 9, i.e., *A* = 3, and the total number of permutations with the possibility of various results with respect to NE in this matrix is 9!/(3!^2^) = 10,080.

Each permutation of the derived payoff matrix is a game in the basic form with at least one NE. The condition for the best mutual response of two opponents (i.e., player *n* and player *m*) with respect to a Nash equilibrium is as follows [6]: Let *x*_*n*_ and *x*_*m*_ be mixed strategies of players *n* and *m*. Then, *x*_*n*_ is the best response to *x*_*m*_ if for *i* *ϵ* *A*_*n*_ *x*_*n*,*i*_ > 0 ⇒ (*π*_*n*_ *x*_*m*_)_*i*_ = max(*π*_*n*_ *x*_*m*_)_*k*_ applies, and *x*_*m*_ is the best response to *x*_*n*_ if for *j* *ϵ* *A*_*m*_ *x*_*m*,*j*_ > 0 ⇒ (*π*_*m*_ *x*_*n*_)_*j*_ = max(*π*_*m*_ *x*_*n*_)_*k*_ applies.

NE can be calculated as linear programming tasks for each matrix permutation. Specific calculations can be performed via labeled polytopes [36, 37]. For example, a 3 × 3 bimatrix game can be geometrically interpreted as two polyhedra in space that represents each player’s individual strategies: *x*_1_, *x*_2_, *x*_3_ for Player 1 and *y*_4_, *y*_5_, *y*_6_ for Player 2. NE can be evaluated as the completely labeled vertices of these polytopes, which ensures that by definition the vertices are found at local maxima that correspond to identical rows and columns within each payoff matrix. The evaluated options represent games with NE on the generated polytope axes in pure strategies, on faces in mixes of two strategies, and for a proportion of payoff bimatrix permutations at the intersection of three faces in space in mixes of three strategies.

NE is found for every game from the complete set of evaluated permutations *V* of a symmetrical game matrix. Every found NE represents a vector of selection of formal strategies. That is, for player *n*, $x_{n,1}...x_{n,A},\sum_{i=1}^{A}x_{n,i}=1$. The resulting values *x*_*n*,*i*_ are counted as contributions to the total degree of equilibrium *E*_*nk*_ of player *n* in a situation *k* that corresponds to the determined NE. In the case of NE in pure formal strategies, the contribution *x*_*n*,*k*_ = 1 is the same for all players. For NE in mixed strategies, the contributions may be *x*_*n*,*i*_ in individual situations (representing NE) for each player.

Each complete set of permutations of the symmetrical matrix (therefore, the game) has weight 1. If there is more than one NE in one game, the values of the counted contributions are divided by the number of determined equilibriums *l*_*E*_. The resulting values of total *E*_*n*,*k*_ are obtained as summary contributions of all *V* of solved games:

$$\begin{array}{c} E_{n,k}=\sum_{V}{\left(\frac{x_{n,k}}{l_{E}}\right)}_{v} \end{array}$$ (2)

Degrees of equilibrium for individual situations and players (therefore, interacting subjects) can be additionally relativized by dividing by a total number of evaluated permutations:

$$\begin{array}{c} p_{n,k}=\frac{E_{n,k}A{!}^{N}}{A^{N}!},\;\sum_{k=1}^{K}p_{n,k}=1 \end{array}$$ (3)

This step results in the expression of the equilibrium solution of the derived game in the form of sets of values of probability *p*_*n*,*k*_, thereby determining NE in the arbitrary permutation of a symmetrical game matrix for *N* interacting subjects in *K* situations.

The values *p*_*n*,*k*_ characterize Nash equilibrium probabilities which could be interpreted as relative degrees of stability for interacting subjects in individual situations. Higher values indicate a preferable compromise of payoff values of interacting subjects in relation to equilibrium and therefore also a higher stability of evaluated situations.

In general contexts, the approach employed can also be validated (for case of two players) using an analogy with a corresponding elementary card game: Each player has *K* cards with various values. In each round of the game, one of the players arranges these *K* cards into a square with *A* cards per side (*A*^2^ = *K*). The other player matches his or her cards to the cards the first player arranged to make prescribed pairs. *K* identical pairs are created each round and no other pairs are permitted. The order of the players alternates between individual rounds. The card pairs’ square distribution is evaluated in each round such that each player is assigned the points of his or her cards or shares of cards which represent NE. The players’ primary effort is to be assigned cards with the highest possible value. However, the players do not know the specific card distribution methods (the payoff bimatrix variations that lead to NE) with the greatest number of these highest cards. The distribution of card pairs in each round is therefore random. If all possible distributions are taken into account, it is possible to determine which cards of each player will win more frequently in the sense of their proportions of the NE found. The game’s total points are therefore determined by the card values distributed to each player and how these cards are prescribed within the *K* defined pairs.

## Example

As an example, an application of the proposed model in the field of plant ecology is described. The example concerns the evaluation of the stability of interactions between the expansive bushgrass *Calamagrostis epigejos* and other plant species in nine environments under various types of management.

The evaluated data were acquired over two seasons in an experiment performed in complete, randomized blocks with three repetitions. The experimental design was based on long-term experiments performed in Central Europe [38, 39]. Each block consisted of nine treatments represented by squares with an area of 9 m^2^ within which various fertilization rates and mowing / no mowing managements were applied: C-cut, C-uncut, N-cut, N-uncut, K-uncut, NP-cut, NP-uncut, NPK-cut, and NPK-uncut. C designates variants with no fertilization, and C-uncut corresponds to squares without any intervention. Nutrients were supplied by applying commercial fertilizers. A community’s response to added nutrients is rapid and typically results in a decrease in species diversity at the local level [40, 41]. During the second season, the relative spatial proportion of *C. epigejos* and the number of other plant species that occurred were evaluated for each square. To eliminate any possible distortion due to the edge effect, data were collected only in the central area of each square.

**Fig 1:**
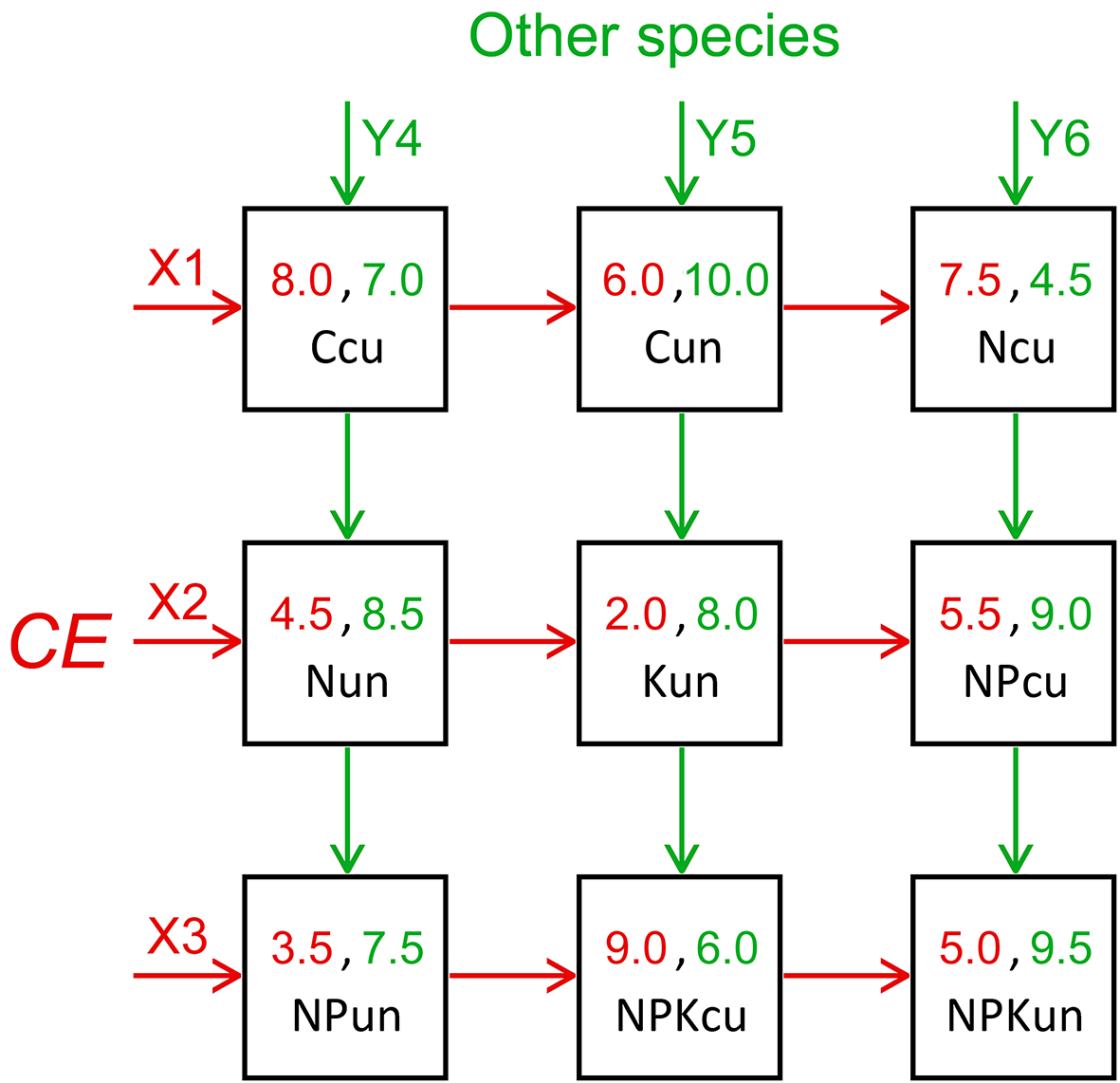
The derived payoff bimatrix: One of the 10,080 permutations. The bimatrix includes abundance values of the bushgrass Calamagrostis epigejos (CE) in multiples of 10% and numbers of the other species that were found (individual abundances of the other species have not taken into account).

For the proposed evaluation method, data were available on the determined abundance of *C. epigejos* and the numbers of other species within the nine management treatments (individual abundances of the otrher species have not taken into account). Subject 1 is bushgrass with a payoff table that expresses its abundance within individual squares in multiples of 10%. Subject 2 consists of other found species, and the payoff table states their determined numbers. All values represent the rounded means (with a minimum increment of 0.5) of each treatment’s three repetitions. The particular payoff values for both subjects are provided in Fig 1. The vector of payoff values is dimension AN, and its additional transformation is unnecessary. Nine pairs of payoff values may be directly distributed into a 3 × 3 symmetrical matrix (Fig 1).

An overview of the total number of NE calculated for all 10,080 payoff bimatrix permutations (i.e., games) is provided in Table 1. NE was most frequently found in pure strategies, whereas in mixes of three formal strategies, NE occurred in fewer than 10% of the evaluated permutations.

**Table 1:** Total Nash equilibrium (NE) found.

Variant	Summary NE in pure strategies		Summary NE in mixes of two strategies		Summary NE in mixes of three strategies		Total summary NE	
	Subject 1 CE	Subject 2 Species	Subject 1 CE	Subject 2 Species	Subject 1 CE	Subject 2 Species	Subject 1 CE	Subject 2 Species
C-cut	360.0	360.0	799.0	654.9	101.0	75.0	1260.0	1089.9
C-uncut	3403.3	3403.3	621.9	548.9	64.8	85.9	4090.0	4038.2
N-cut	0.0	0.0	198.5	390.1	48.1	86.1	246.7	476.2
N-uncut	72.0	72.0	353.8	446.2	81.9	76.5	507.7	594.7
K-uncut	0.0	0.0	254.6	198.0	82.1	58.3	336.7	256.3
NP-cut	973.3	973.3	602.5	586.8	76.7	84.2	1652.5	1644.4
NP-uncut	0.0	0.0	265.5	259.6	84.1	68.5	349.6	328.1
NPK-cut	0.0	0.0	352.0	372.9	68.2	61.7	420.2	434.6
NPK-uncut	612.3	612.3	534.9	525.3	69.5	80.0	1216.7	1217.6
Total	5421.0	5421.0	3982.7	3982.7	676.3	676.3	10080.0	10080.0

**Fig 2:**
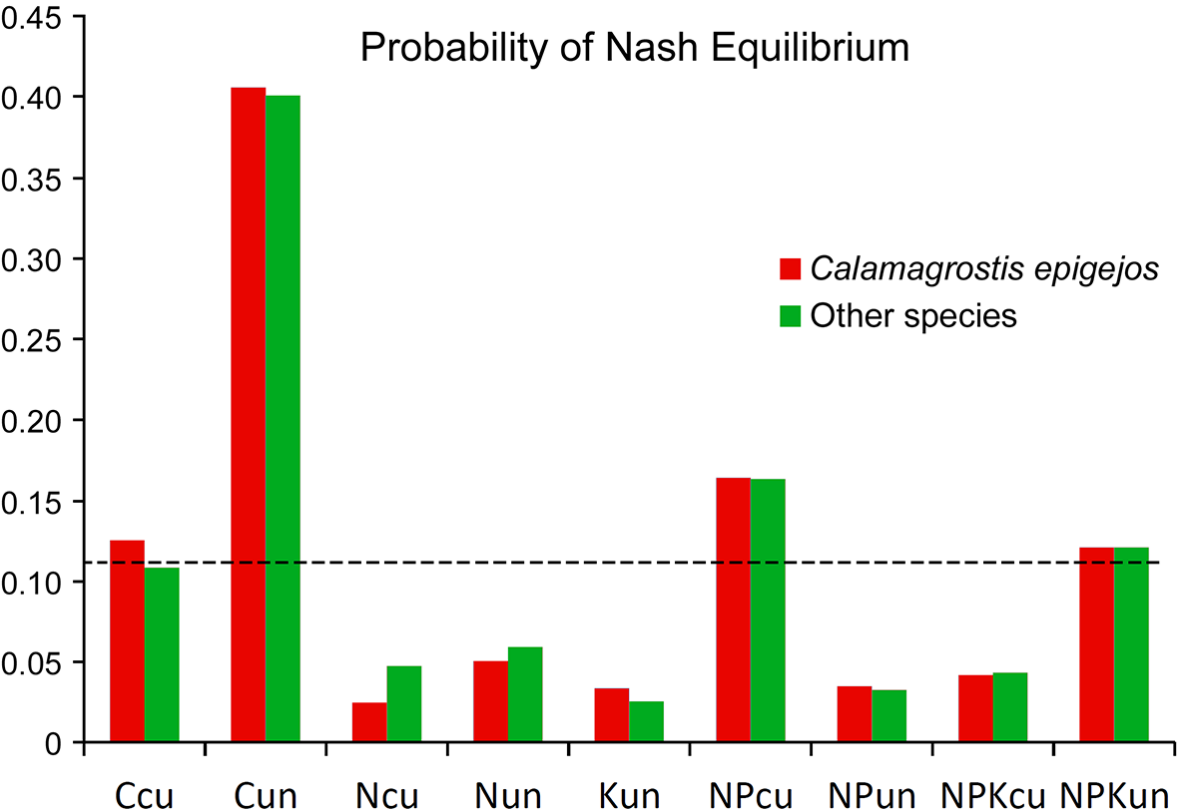
Obtained Nash equilibrium probabilities for individual management treatments. The dotted line is the expected value of 1/9 that corresponds to a uniform distribution of NE probability.

The resulting NE probabilities *p*_*n*,*k*_ for the individual management variants are shown in Fig 2 For each of the nine variants, we can define an expected value of 1/9 corresponding to a uniform distribution of NE probability. Within the context of the approach that was used, this value can be viewed as an indifferent state.

**Fig 3:**
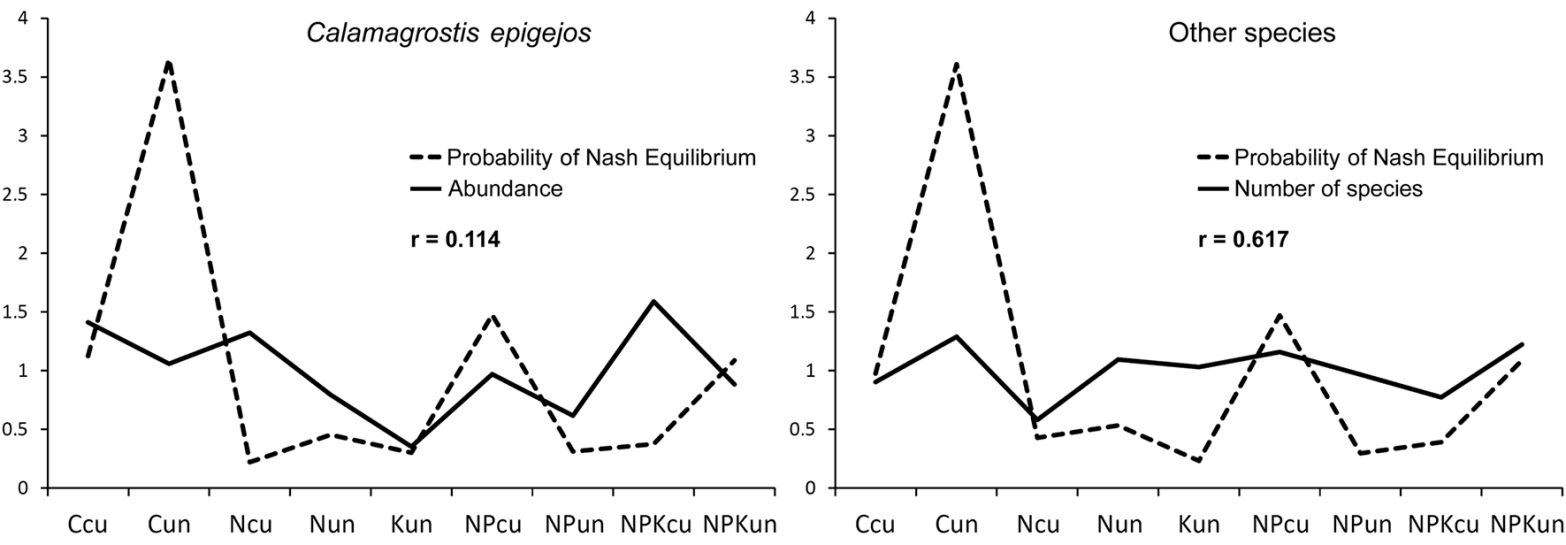
Comparison of the trend of NE probabilities for bushgrass abundance and number of other species. Presented trends are normalized to the mean value of one.

The results show that the application of the proposed concept offers the possibility of solving the problem of equilibrium optimum, i.e., the best compromise among payoff values, which are optimal for each interacting subject. The setting is not trivial, and the maximum payoff is achieved in both subjects in different situations. For *C. epigejos*, NPK-cut is an optimal situation. The other species achieve their highest numbers in the C-uncut situation. The evaluation of the initial payoff values according to the proposed concept unambiguously results in a preferred maximum number of other species, and thus a significantly higher probability of NE occurrence *p*_*n*,*k*_ results, which can be interpreted as a degree of stability (Fig 2). This result approximately matches common assumptions. That is, the highest degree of stability is achieved in a situation without interference, and this stability corresponds to the number of occurring species; therefore, corresponds to biodiversity and not to the abundance of expansive bushgrass. This fact is not only demonstrable with respect to the maximum obtained value *p*_*n*,*k*_. The result of the proposed concept is a continuous distribution of the degree of stability of the evaluated situations. The correlation of this dependence with the set of initial payoff values unambiguously results in the beneficial occurrence of other species—*r* = 0.617 against *r* = 0.114 for *C. epigejos* abundance. That is, stability corresponds to biodiversity (Fig 3).

The determinant factor with respect to the result is the occurrence of NE in pure formal strategies such that the calculated distribution of the degree of stability is not significantly different for both subjects. Therefore, it is impossible to deduce a recommendation regarding the type of management that might be more beneficial for the stability of occurrence of other species compared with the abundance of *C. epigejos*. However, the results of the evaluation may represent a contribution to research on the spread of this expansive grass. Based on the evaluation, a summary is possible: there are types of management that result in a more remarkable elimination of the occurrence of *C. epigejos* in plant communities, e.g., K-uncut. However, the assumed degree of stability of these management types is low compared with the non-interference situation, and these states may not be sustainable in the long term.

## Discussion

The proposed model of evaluation of the occurrence of NE probability for a set of variant situations and subjects interacting in these situations can be considered to be a solution of a game with vector payoffs. The equilibrium concept in zero-sum matrix games with vector payoffs was developed by Shapley [42]. Variable payoffs may be considered as randomly distributed in commonness, therefore as values with random deviation. Harsányi [24] designed a model of purification of mixed equilibrium that enables a deterministic solution of equilibrium in a game with randomly distributed payoffs. In this paper, we have addressed a situation in which the quantitative results of interactions of the involved subjects (i.e., players) are well known, as are the payoffs in individual situations. However, there is no information on player strategy, which results in a particular limitation of the position of individual situations (linked with player payoffs) in the game matrix. We assume that this uncertainty can be revolved using a statistical set of all permutations of an evaluated situation in a derived symmetrical game matrix. NE is explicitly searched for in every permutation, and its occurrence (in pure or mixed strategies) is gradually counted for individual players in evaluated situations. Thus, our model is simple: the calculation of the probability distribution of NE occurrence does not require complicated mathematical generalization. Considering the established concept of a game with vector payoffs, in our model, all of the vectors in the game matrix are identical. In every solved step (i.e., permutation), the selection of individual payoff values is unambiguously determined by the permutation rules of the evaluated situations (linked with payoffs) in the game matrix.

The presented example of the application of the proposed concept of stability evaluation in datasets of a given type is a simple one with an unambiguous, easily substantiated result. It is obvious why the highest probability of NE occurrence results in the maximum occurrence of other species (in the situation without interference C-uncut) compared with the maximum abundance of *C. epigejos* (in NPK-cut) with the degree of equilibrium being low. The factor that determines the resulting distribution of the degree of equilibrium is NE occurrence in pure formal strategies for the given set of payoff values (Table 1). It is understandable from the initial payoff values that the abundance of *C. epigejos* achieves relatively higher values in a situation that includes the maximum occurrence of other species (i.e., C-uncut). Among the eight other situations, five display a lower abundance value of *C. epigejos* than occurs in situation C-uncut. Thus, the probability of finding NE in purely formal strategies for the situation of the maximum occurrence of other species (i.e., C-uncut) in any permutation of symmetrical game matrix 3 × 3 is 20:56 (0.36), which agrees with the obtained result (Fig 2) (this outcome also includes the lesser NE frequency that results from mixed formal strategies). In contrast, the situation with the maximum abundance of *C. epigejos* (i.e., NPK-cut) includes the smallest number of other species of all nine situations. Thus, the probability of NE occurrence in pure formal strategies is zero for this situation, which is documented by the results presented in Table 1.

Of course, the proposed procedure of stability evaluation in the system represented by the determined payoff values *N* of interacting subjects in *K* situations can be used to evaluate more complex interactions than those included in the presented example. For example, the procedure may be applied to interactions of more subjects with an ambiguous probability preference of equilibrium maxima. In such cases, the obtained stability distribution can be predominantly determined by NE found in mixed formal strategies, and thus, the values of NE probability will be more clearly different for the interacting subjects of the individual situations. Naturally, stability distribution is not required to correlate in individual subjects with their initial payoff values.

The significant feature of the proposed concept is that the result—the evaluation of the degree of stability based on the NE principle—is guaranteed for an arbitrarily complex interactive system. Every permutation of the derived matrix of any dimension *N* can be considered to be a payoff table of a game that has at least one NE. The full statistical set of NE occurrence in the permutations of the game matrix is always available for the evaluation of the distribution of the degree of stability.

The proposed approach is only based on the comparison of measurable or observable values of quantitative parameters without the inclusion of specific internal factors that determine the complex behavior of the ecosystem. The interpretation of a larger probability of NE occurrence as a higher stability of ecological interaction is an assumption that often is not applicable. In addition, the degree of stability defined in this way is only relative. It is larger or smaller only compared with the results obtained for other evaluated situations.

**Fig 4:**
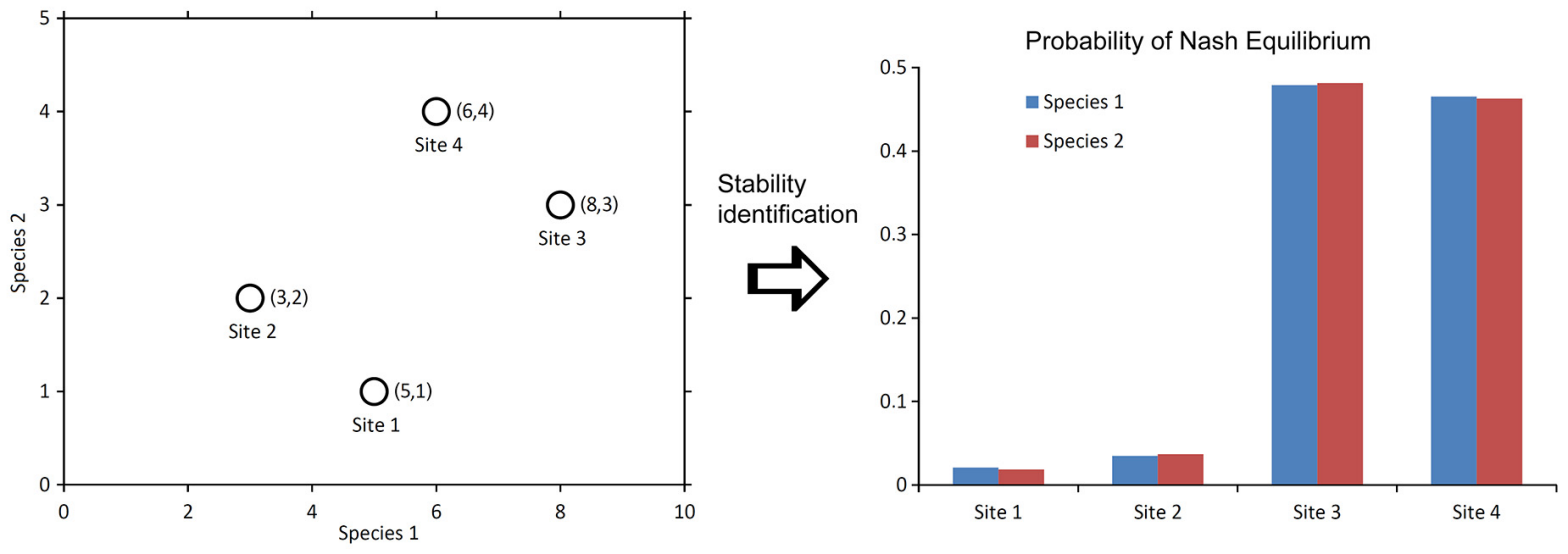
Evaluation of degree of stability expressed as the Nash Equilibrium probability for simple numerical example of two species observed at four sampling sites (mentioned in Chapter 4 of Legendre and Legendre Numerical Ecology [35]).

However, approaches based on game theory and NE application are often used in ecology, particularly in evolutionary ecology, as noted in the introduction. The proposed concept, which is exclusively derived from the stochastic game model, can have its foundation in the context of frequent exploitation of game theory principles for solving ecological problems. Out of all of the supposed applications of varying complexity, the use of proposed approach can be demonstrated on general numerical example of two species observed at four sampling sites, cited in the introduction [35]. It is a very simple example of a multidimensional quantitative data set. The statistical evaluation primarily leads to the covariance matrix and the result is an information on the degree of similarity or differences of descriptors of behavior in the set of selected sites. Our proposed model assesses the situation in a completely different way. The result shows the distribution of degrees of stability of the descriptors in the set of individual sites. We assume, therefore, that we are able to determine more stable and less stable sites (Fig 4).

Relative ecological stability could be evaluated in this way, particularly through the application of our approach to more extensive datasets that reflect, e.g., the ecological parameters of a larger number of landscape elements. In addition, the evaluation of NE probability of occurrence could suitably complement the standard statistical analysis of data of this type. The proposed concept of using NE to solve settings formalized in the Pareto optimality scheme is surely applicable in many contexts including multicriteria analysis of complex ecological and other systems.
